# Case report of rare right intraventricular thrombus: a diagnostic and anticoagulation management challenge

**DOI:** 10.1093/ehjcr/ytaf501

**Published:** 2025-10-03

**Authors:** Yangsin Lee, Shogo Shimada, Minoru Ono

**Affiliations:** Department of Cardiovascular Surgery, Graduate School of Medicine, The University of Tokyo, 7-3-1 Hongo, Bunkyo-ku, Tokyo 113-8655, Japan; Department of Cardiovascular Surgery, Graduate School of Medicine, The University of Tokyo, 7-3-1 Hongo, Bunkyo-ku, Tokyo 113-8655, Japan; Department of Cardiovascular Surgery, Graduate School of Medicine, The University of Tokyo, 7-3-1 Hongo, Bunkyo-ku, Tokyo 113-8655, Japan

**Keywords:** Right ventricular thrombus, Cardiac tumour, Antiphospholipid syndrome, Anticoagulation, Case report

## Abstract

**Background:**

Antiphospholipid syndrome (APS) is a rare autoimmune disorder characterized by an increased risk for both arterial and venous thrombosis. This case of a right intraventricular thrombus mimicking a cardiac tumour highlights the complexities of anticoagulation management in APS.

**Case summary:**

A right intraventricular mass was incidentally discovered in a 54-year-old woman undergoing assessment for an unexplained cerebral stroke. Imaging revealed a 27 × 15 mm pedunculated mass in the right ventricle without atrial fibrillation, intracardiac shunt lesion, or deep vein thrombosis. The patient was initially started on a direct oral anticoagulant (DOAC), but the intracardiac mass did not change in size, prompting a referral for further management. Due to the large size and high mobility of the mass, and the associated risk of pulmonary embolism, surgical resection of the mass was performed. Histopathologic examination revealed the mass to be an organized chronic thrombus. Post-operatively, the patient continued treatment with a DOAC but experienced recurrent cerebral strokes requiring 4 months of rehabilitation, which delayed the definitive diagnosis of APS. Anticoagulation therapy was changed to therapeutic warfarin thereafter.

**Discussion:**

Diagnosing a right intraventricular thrombus is particularly challenging if it is the first clinical venous thrombotic event associated with APS. This case emphasizes the importance of considering APS in patients presenting with unexplained arterial and venous thrombosis. Warfarin is preferred over DOACs unless the diagnosis of APS is ruled out, even if the thrombus is completely removed.

Learning points

- A right intraventricular thrombus can be an uncommon but significant manifestation of antiphospholipid syndrome (APS).
- Anticoagulation therapy, typically with warfarin, should be initiated early in APS patients to prevent recurrent thrombus formation, though a definitive diagnosis takes at least 3 months to obtain.

## Introduction

Antiphospholipid syndrome (APS) is a systemic autoimmune disease with various vascular and obstetric manifestations.^1^ Antiphospholipid syndrome has an annual incidence of approximately two cases per 100 000 population and an estimated prevalence of 50 per 100 000 population.^2^ Common clinical features include deep vein thrombosis (DVT) as venous thromboembolism (VTE), ischaemic stroke as arterial thrombosis, and pregnancy losses; simultaneous arterial and venous thrombosis as the first APS clinical manifestation, however, is extremely rare.^3^ This case of a right intraventricular mass highlights the diagnostic and management challenges posed by the rarity and complexity of this condition.

## Summary figure

**Table:** 

Date	Event
3 years prior to presentation	Uterine leiomyomas and associated anaemia (lost to follow-up)
4 weeks prior to presentation	Ischaemic stroke. Echocardiography showed a mass in the right ventricle with no shunt lesion or left atrial appendage thrombus. Duplex sonography ruled out DVT
3 weeks prior to presentation	Start of anticoagulation with apixaban (no change in size of the intraventricular mass). Computed tomography showed a large pelvic mass: histological examination revealed no malignancy
2 weeks prior to presentation	Cardiac magnetic resonance imaging (MRI) showed a pedunculated mass in the right ventricle suggestive of a cardiac tumour
Upon presentation	Successful surgical resection of the tumour (confirmed as organized thrombus on histology) and left atrial appendage
10 days after surgery	Hospital discharge with edoxaban
2 months after surgery	IgG anticardiolipin antibody-positive, decision to continue edoxaban by the rheumatology team (awaiting another antiphospholipid antibody test at least 12 weeks apart)
4 months after surgery	Recurrent ischaemic stroke requiring 4 months of rehabilitation, anticoagulant changed to heparin
5 months after surgery	Anticoagulant changed to warfarin with international normalized ratio (INR) 1.5–2 considering vaginal bleeding
7 months after surgery	DVT in the left upper and right lower limb
8 months after surgery	Definitive diagnosis of APS. INR target increased to 2–3. Echocardiography showed no residual intracardiac mass
9 months after surgery	Discharge from rehabilitation hospital. The gynaecology team considering pelvic mass resection

## Case presentation

**Figure 1:**
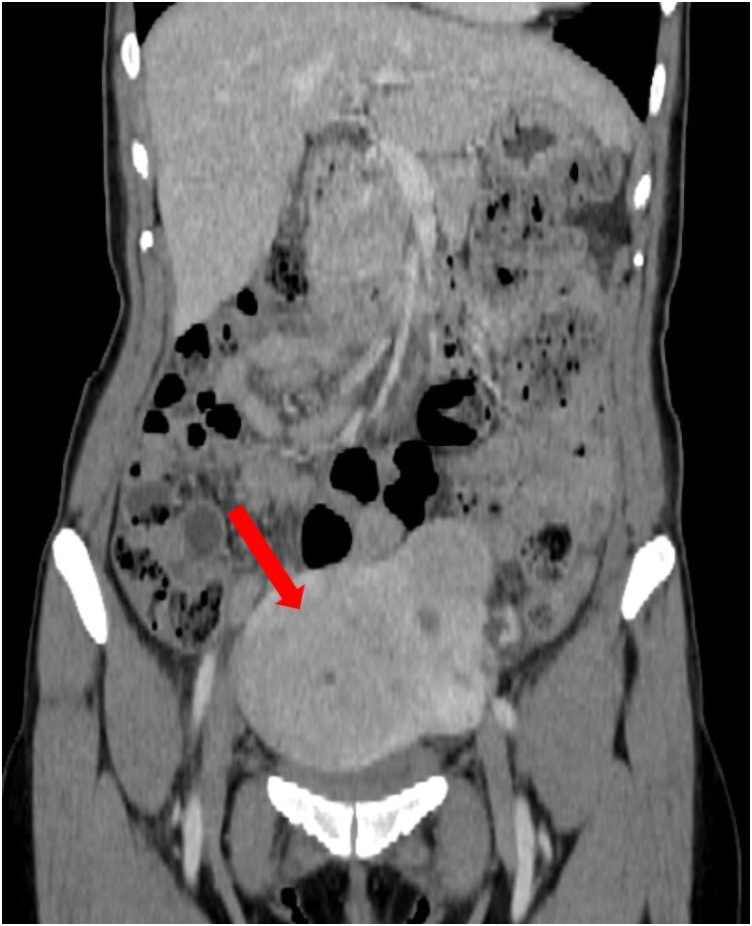
Computed tomography image showing a large pelvic mass measuring approximately 10 cm in size.

A 54-year-old woman was admitted to the referring hospital with dysarthria and dysphagia and diagnosed with multiple small cerebral infarcts by MRI. She had a 3-year history of untreated uterine leiomyomas and anaemia, but no pregnancy history. She was a non-smoker with no history of hyperlipidaemia, diabetes, or hypertension. On physical examination, there was no heart murmur, extremity oedema, or elevation of jugular venous pressure, but a palpable uterus was noted. Electrocardiography showed normal sinus rhythm. Laboratory tests showed haemoglobin 7.5 g/dL (11.6–14.8 g/dL), D-dimer 8.4 µg/mL (<1.0 µg/mL), and CA125 786 U/mL (<35 U/mL); all other haematological and biochemical parameters were within normal limits. Transthoracic echocardiography revealed a highly mobile mass in the right ventricle. Subsequent transoesophageal echocardiography demonstrated a 27 × 15 mm pedunculated mass near the tricuspid valve, without deterioration of valvular or ventricular function (see Supplementary material online, *Video 1*). In addition, there was no evidence of left ventricular apical or left atrial appendage thrombus, and a patent foramen ovale was also not detected on bubble study with Valsalva manoeuvre, a potential source of paradoxical embolism. Colour Doppler and duplex-scan ultrasonography ruled out DVT. Apixaban, a direct oral anticoagulant (DOAC), 10 mg twice daily, was initiated for 1 week after the mass was detected, but the size did not change, favouring a diagnosis of primary or secondary cardiac tumour over a thrombus. Whole-body computed tomography revealed a large (∼10 cm) pelvic mass (*Figure 1*), but no other malignant lesions. Cardiac MRI confirmed a right intraventricular mass attached to the anterior wall (see Supplementary material online, *Video 2*). Although poorly defined on T1- and T2-weighted sequences, the signal intensity resembled myocardium, making differentiation between thrombus and tumour difficult. Given the size and mobility of the mass and the high risk of pulmonary embolism (PE), surgical management was considered necessary, and the patient was referred to our cardiac surgery centre.

**Figure 2:**
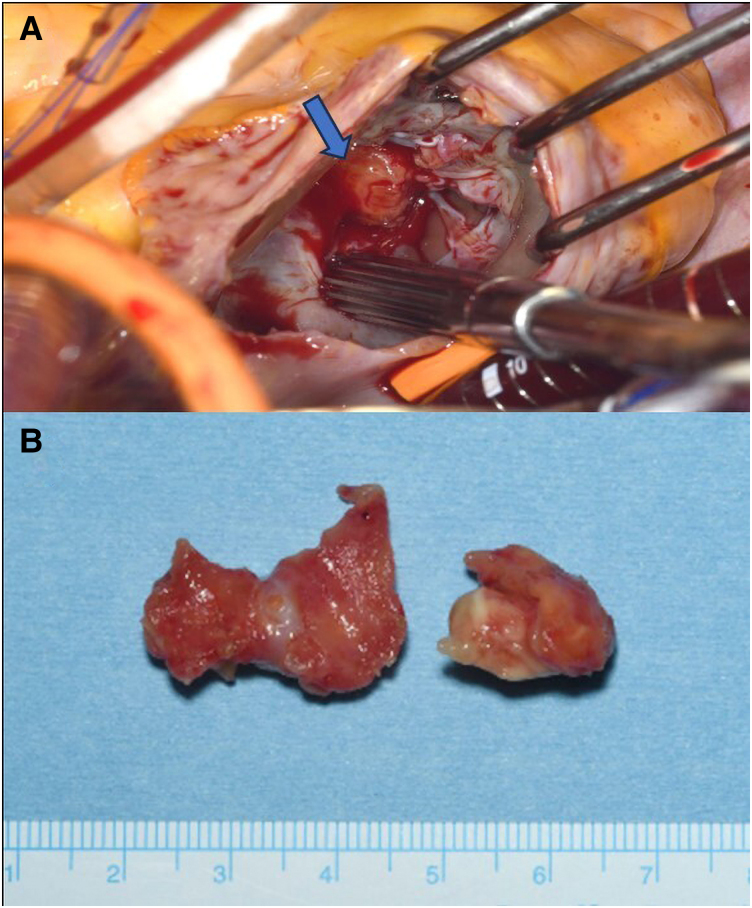
(*A*) Intraoperative view. Right intraventricular mass, confirmed through a right atriotomy, was attached to the interventricular septum, anterior wall, and posterior leaflet chords of the tricuspid valve. (*B*) The surgically removed mass comprised two parts.

**Figure 3:**
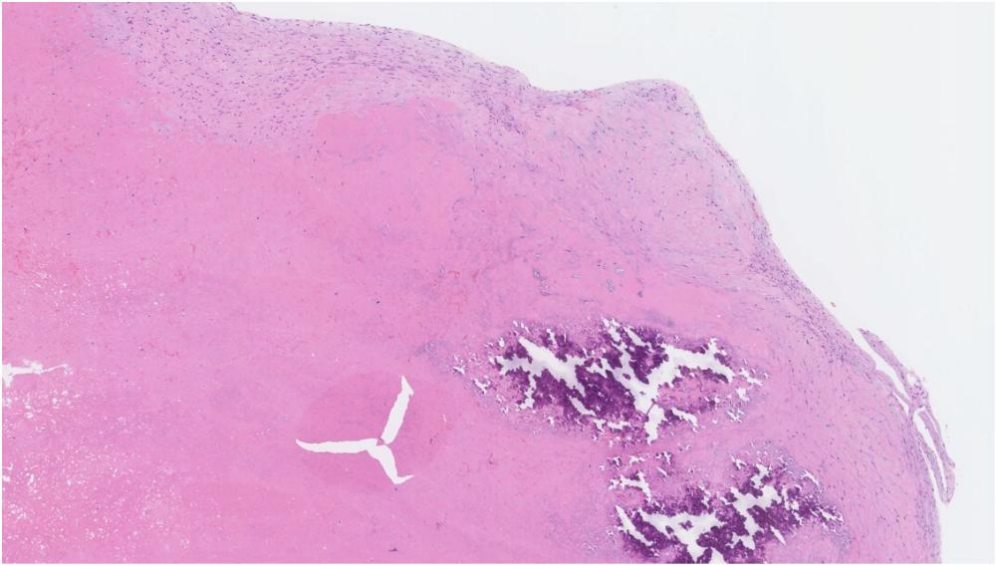
Histological examination showing an organized thrombus composed mainly of fibrin with focal calcification (haematoxylin and eosin stain, 50× field).

On presentation to our centre, she had recovered well from her cerebral infarcts without neurological deficits and remained in sinus rhythm. Histological examination of the pelvic mass at the referring hospital revealed no malignancy, suggesting uterine leiomyomas and/or adenomyosis. Given the risk of PE, we performed urgent open-heart surgery to remove the intracardiac mass and left atrial appendage through a median sternotomy with standard bicaval cardiopulmonary bypass and antegrade cardioplegia with aortic cross-clamping. A right atriotomy (*Figure 2A*) revealed a right intraventricular mass comprising two parts: a 15 mm lesion attached to the interventricular septum and adhering to a posterior leaflet chord of the tricuspid valve and a 25 mm lesion adhering to the anterior wall and a posterior chorda tendinea (*Figure 2B*). The entire mass was easily resected. Histology revealed an organized thrombus comprising mainly fibrin with focal calcification (*Figure 3*); anticoagulation with edoxaban 30 mg daily was started to prevent recurrence. Post-operative recovery was uneventful, and the patient was discharged home 10 days after surgery.

Testing indicated a low likelihood of cardioembolic stroke, with no evidence of left heart thrombus, patent foramen ovale, or atrial fibrillation. The aetiology of the right intraventricular thrombus remained uncertain as no thrombus was detected in the lower extremities or pelvic veins. These findings suggested less typical aetiologies, such as hypercoagulable states. Screening laboratory evaluation for antiphospholipid antibodies (aPL) in the outpatient setting 2 months after surgery revealed an elevated IgG anticardiolipin antibody (aCL) titre without lupus anticoagulant or antibeta 2 glycoprotein I antibodies. The patient was referred to rheumatology and edoxaban was continued according to VTE guidelines^4,5^ until the diagnosis of APS was confirmed. At 4 months after surgery, she experienced another ischaemic cerebral infarction. Given the high suspicion for APS, the anticoagulant was changed from a DOAC to heparin and subsequently to warfarin. Due to her menorrhagia, the warfarin dose was adjusted to maintain a target INR of 1.5–2.0. She required 4 months of rehabilitation, during which she developed DVTs in the left upper and right lower limbs. A definitive diagnosis of primary APS was established 8 months after surgery based on a persistent moderately elevated IgG aCL titre, and warfarin was adjusted to achieve a therapeutic INR of 2.0–3.0.

## Discussion

This case of a rare intracardiac thrombus highlights the challenges in diagnosing APS and determining the optimal anticoagulation strategy. Only a few cases of surgical resection of a right intraventricular thrombus with APS are reported.^6^ The present case is the first in which simultaneous cerebral stroke and right intraventricular thrombus occurred as the first clinical event in a patient with APS.

Considering our patient’s age, the mass location, and the underlying large pelvic mass with high tumour marker levels, the pre-operative differential diagnosis included primary tumours (papillary fibroelastoma, lipoma, haemangioma, and blood cysts), secondary tumours (cardiac metastases), vegetation (non-bacterial thrombo-endocarditis), and, less likely, thrombus.^7^ Cardiac MRI helps differentiate between thrombus and tumours,^8^ but variable T1- and T2-weighted signals based on thrombus age complicate diagnosis.^8^ The large pelvic mass further complicated the diagnosis; although cervical and endometrial cytology were negative for malignant cells, benign metastasis of the leiomyoma to the heart^9^ was also considered. Antiphospholipid syndrome was not suspected before surgery; a history of pregnancy morbidity might have raised earlier suspicion.

Compared with left-sided cardiac thrombus, there are no established guidelines for optimal management of right-sided cardiac thrombus. The treatment options, including anticoagulation, thrombolysis, and surgical intervention, remain under debate.^10^ Because of the patient’s large (>1 cm), highly mobile mass,^11^ we selected surgical resection to prevent PE and diagnosed her post-operatively. According to guidelines, a DOAC was administered post-operatively to prevent PE recurrence.^4,5^ Screening laboratory evaluation was conducted in the outpatient setting according to the current APS criteria, which require at least two positive aPL tests performed at least 12 weeks apart, along with clinical features for a definitive diagnosis.^12^ No clear guidelines address management of aPL-positive patients with prior thrombosis awaiting definitive APS diagnosis. After initial aPL testing, a multidisciplinary team, including rheumatology and gynaecology, decided to continue DOAC per VTE guidelines for patients without APS,^4,5^ considering the patient’s bleeding/thrombosis risk. Definitive diagnosis was delayed, however, due to recurrent cerebral infarctions requiring long-term rehabilitation.

Although the treatment regimen for APS varies widely, warfarin (target INR of ≥2–3) is recommended over a DOAC or low-dose aspirin only for patients with APS presenting with the first arterial or venous thrombosis, considering the individual’s bleeding/thrombosis risk.^1^ For patients with recurrent arterial or venous thrombosis despite adequate treatment, adding low-dose aspirin or increasing the target INR may be considered.^1^ In our patient, menorrhagia associated with uterine fibroids interfered with adequate anticoagulation or the addition of antiplatelets, which might lead to recurrent stroke and DVT. Eventually, however, therapeutic warfarin was given in anticipation of gynaecological surgical treatment.

## Conclusions

This case highlights the diagnostic and management challenges of a right intracardiac mass. Although rare, an APS-associated thrombus should be considered in the differential diagnosis for patients presenting with an intracardiac mass. Even after complete thrombus removal, warfarin is preferred over DOACs to prevent recurrent thrombus formation unless APS is ruled out.

## Lead author biography


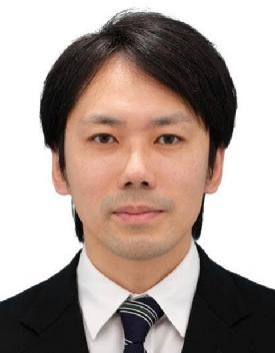


Dr Yangsin Lee is a dedicated cardiac surgeon at the University of Tokyo, Japan. After completing his PhD in 2019, he pursued a clinical fellowship in Cardiothoracic Surgery at John Hunter Hospital in Australia. He has a strong interest in heart failure, mechanical circulatory support, and medical research.

## Supplementary Material

ytaf501_Supplementary_Data

## Data Availability

Data supporting this article will be shared upon reasonable request to the corresponding author.

## Supplementary material

**Consent:** Informed consent was obtained from the patient in accordance with the COPE guidelines.

**Funding:** None declared.
